# Association between physical activity changes and incident myocardial infarction after ischemic stroke: a nationwide population-based study

**DOI:** 10.1186/s12889-024-18724-2

**Published:** 2024-05-06

**Authors:** Dae Young Cheon, Kyung do Han, Yeon Jung Lee, Jeen Hwa Lee, Myung Soo Park, Do Young Kim, Jae Hyuk Choi, Sook Jin Lee, Kyung-Ho Yu, Seongwoo Han, Sunki Lee, Minwoo Lee

**Affiliations:** 1 Division of Cardiology, Department of Internal Medicine, Dongtan Sacred Heart Hospital, Hwaseong, Korea; 2 Department of Statistics and Actuarial Science, Soongsil University, Seoul, Korea; 3 Department of Neurology, Hallym University Sacred Heart Hospital, Anyang, Korea; 4 Division of Cardiology, Department of Internal Medicine, Korea University Guro Hospital, Seoul, Korea

**Keywords:** Ischemic stroke, Myocardial infarction, Physical activity, Lifestyle

## Abstract

**Background:**

The impact of changes in physical activity after ischemic stroke (IS) on the subsequent myocardial infarction (MI) risk is not fully understood. We aimed to investigate the effects of changes in physical activity on the risk of MI after acute IS using data from the Korean National Health Insurance Services Database.

**Methods:**

224,764 patients newly diagnosed with IS between 2010 and 2016 who underwent two serial biannual health checkups were included. The participants were divided into four categories according to changes in their physical activity: persistent non-exercisers, new exercisers, exercise dropouts, and exercise maintainers. The primary outcome was a new diagnosis of incident MI. Multivariable Cox proportional models were used to assess the effects of changes in exercise habits on the risk of MI.

**Results:**

After a median of 4.25 years of follow-up, 6,611 (2.94%) MI cases were observed. After adjusting for confounders, new exercisers and exercise maintainers were significantly associated with a lower risk of incident MI than persistent non-exercisers (aHR, 0.849; 95% CI, 0.792–0.911; *P*-value < 0.001; and aHR, 0.746; 95% CI, 0.696–0.801; *P*-value < 0.001, respectively). Effects were consistent across sexes, more pronounced in those > 65 years. Notably, any level of physical activity after stroke was associated with a reduced MI risk compared to no exercise.

**Conclusions:**

In this nationwide cohort study, commencing or sustaining physical activity after an IS corresponded to a diminished likelihood of subsequent MI development. Advocating physical activity in ambulatory stroke survivors could potentially attenuate the prospective risk of MI.

**Supplementary Information:**

The online version contains supplementary material available at 10.1186/s12889-024-18724-2.

## Abstract (key message)

**What is New?**

• Initiating or maintaining regular physical activity following an ischemic stroke (IS) diagnosis significantly reduces the risk of myocardial infarction (MI), regardless of physical activity intensity, age, or sex.

• This large-scale cohort study emphasizes the crucial role of physical activity in post-stroke care and secondary prevention, offering a novel perspective on the relationship between physical activity and post-IS cardiovascular outcomes.

**What are the Clinical Implications?**

• Healthcare practitioners should prioritize promoting physical activity as an integral component of post-stroke care for older adults, aiming to reduce MI risk.

• The findings underscore the importance of regular physical activity for both IS and MI prevention and highlight the potential for improved patient outcomes through lifestyle modifications.

**Supplementary Information:**

The online version contains supplementary material available at 10.1186/s12889-024-18724-2.

## Background

Stroke and myocardial infarction (MI) account the leading cause for global death rates [1]. Patients who have suffered an ischemic stroke (IS) often experience MI, owing to the considerable similarities in the pathophysiological traits and risk factors associated with these two conditions [2–4]. MI incidence following IS varies across studies but is generally high, with incidence rates ranging from 2–4.8%2, 5–7]. Therefore, MI prevention following IS occurrence is a critical issue. Although secondary prevention strategies for MI and IS require guideline-based medical interventions, including appropriate antithrombotic treatment and risk factor management, lifestyle modifications remain crucial to the preventative measures [8, 9].

Regular physical activity is recommended for primary and secondary disease prevention to mitigate the impact of cardiovascular diseases [10, 11]. The American Heart Association/American Stroke Association recommends that individuals who experience IS should engage in low- to moderate-intensity physical activity and reduce sedentary behavior as part of their secondary prevention strategies [12]. A study demonstrated that engaging in physical activity before experiencing IS event can significantly reduce overall mortality rates following the event [13]. Additionally, regular physical activity and minimization of sedentary behavior reduce the incidence of IS, MI, and cardiovascular disease (CVD) related deaths [14–16]. Previous studies have examined how physical activity before or after IS affects the risk of MI; however, it remains unclear whether changes in physical activity behavior after an IS diagnosis influence the development of MI after IS.

Consequently, this study aimed to evaluate the effect of changes in physical activity on MI incidence following IS using data available in the Korean National Health Insurance Service Database (K-NHID) and investigate whether physical activity intensity influences the results, thereby providing a more robust understanding of the relationship between physical activity and post-IS cardiovascular outcomes.

## Methods

### Data source and study population

This nationwide cohort study utilized data from the K-NHID of the Korean National Health Insurance Service (K-NHIS). Detailed information on the K-NHID, including the processing and management of the claims database, has been extensively documented in previous studies [17]. The database includes the entire population of South Korea, which solely comprises East Asian populations. The advantages and applicability of the K-NHID, including the provision of different blood tests, demographic results, and socioeconomic status, are well documented compared with other databases. The utilization of the K-NHID is contingent upon receiving approval for the study protocols from both the government’s official review panel and the medical institution’s review board. Particularly, this study was approved by the Institutional Review Board (IRB) of Dongtan Sacred Heart Hospital (IRB number: HDT 2022-04-002). Participants undergoing national health checkups provided written informed consent for their data to be used in this research. All the study procedures were performed in accordance with the principles of the Declaration of Helsinki. All medical statistics meet the criteria of a checklist for statistical assessment of medical papers (the CHAMP statement) [18].

**Fig. 1:**
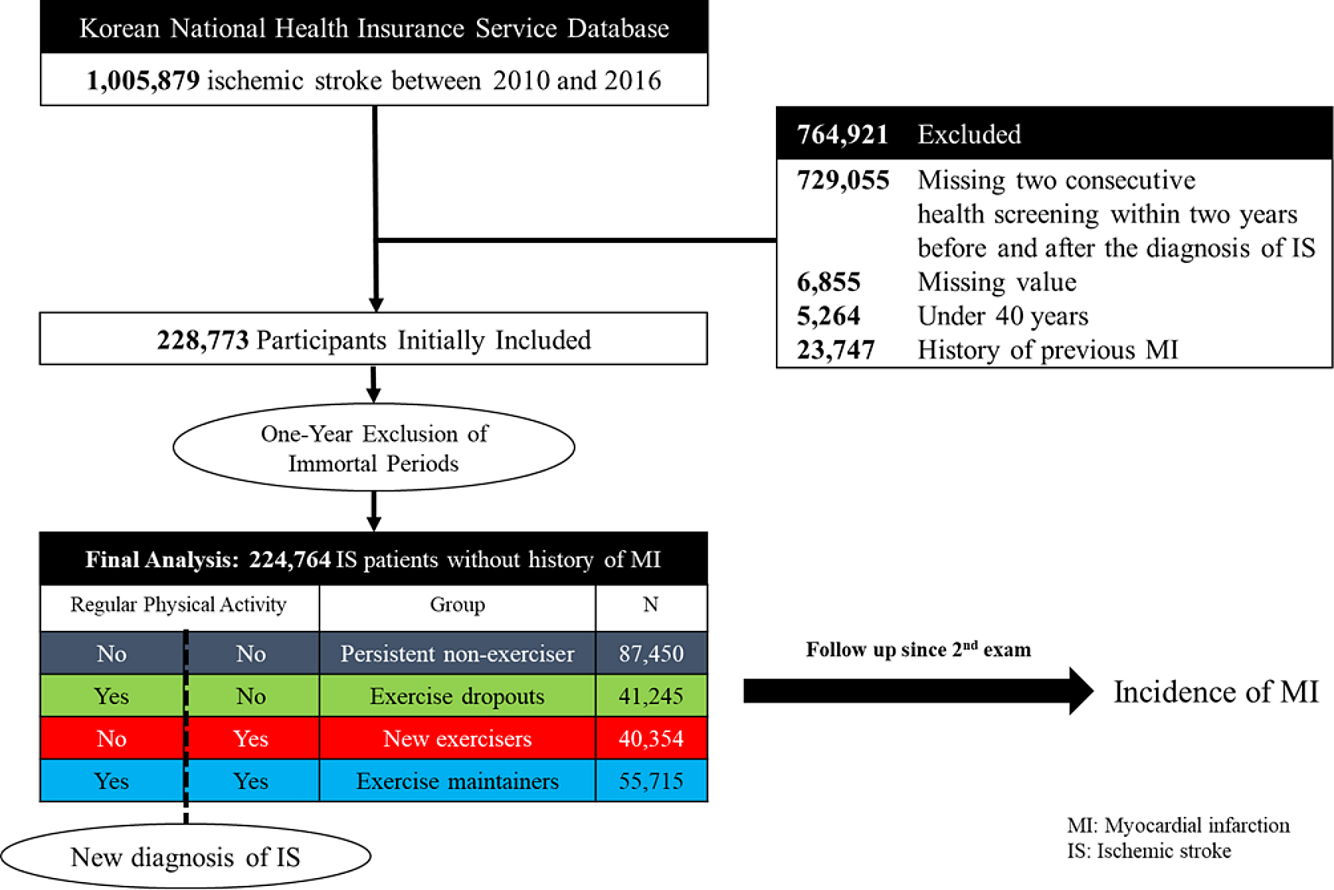
Flowchart of the selection of subjects, definition of exercise habit status and the study protocol

We included 1,005,879 patients diagnosed with first-ever acute IS between 2010 and 2016. The identification of IS was based on ICD-10 codes I63,64 and was only applicable to patients who underwent either brain computed tomography (CT) or brain magnetic resonance imaging (MRI) during admission. This approach has been authenticated in numerous previous studies and is regarded as a reliable method for determining stroke [19–22]. Furthermore, we excluded patients who did not undergo a national biannual health checkup within 2 years before and after the index stroke, resulting in the remaining 264,639 patients. Since national health checkups are conducted exclusively in ambulatory settings, participants who undergo these checkups post-stroke are likely to be survivors with milder forms of the condition. These individuals are at least ambulatory and capable of completing structured questionnaires. We also excluded participants who had missing values in their questionnaires, were below the age of 40, or had a history of MI. Previous MI history was defined using at least one I21-22 code claim. To eliminate immortal bias, we further excluded patients who had at least one I21-22 code claim within a 1-year lag period, leaving 224,764 patients for the final analysis (Fig. 1). The patient cohort was followed up until the end of December 2019, with the most extended follow-up period being 9 years. There were no follow-up losses except for death and emigrations. This research was done without direct patient involvement.

### Main exposure: physical activity change

Participants self-reported their lifestyle behaviors through a questionnaire administered during regular health checkups before and after the diagnosis of IS. The questionnaire included detailed information on the intensity and frequency of the participants’ physical activity. This questionnaire was derived from the International Physical Activity Questionnaire, a tool created by the World Health Organization and adapted to Koreans by Oh et al. [23], and its validity has been confirmed in other studies [24]. The physical activity segment comprised three questions probing the weekly frequency of light, moderate, and vigorous physical activity recently undertaken by the participants. Activities such as slow walking or carpet sweeping for over 30 min were categorized as light-intensity physical activity. Leisure cycling, brisk walking, or tennis doubles playing for over 30 min were classified as moderate-intensity physical activity. Vigorous-intensity physical activity involved running, climbing, swift bicycling, or aerobics for over 20 min. Regular physical activity was defined as participation in moderate or vigorous physical activity at least once weekly. We divided the regular physical activity group based on energy consumption using metabolic equivalents of tasks (METs) to assess how energy expenditure affects MI incidence. Light-, moderate-, and vigorous-intensity physical activities were assigned values of 2.9, 4.0, and 7.0 METs [25], respectively, to estimate the energy consumption [25]. Total energy expenditure was calculated as the sum of each physical activity’s frequency and minimum duration multiplied by the METs. We divided the total energy expenditure into two categories using two different methods. Method was based on a cutoff of 1,000 MET-min, distinguishing between intensive physical activity [26].

The study participants were divided into four groups as follows: (1) persistent non-exercisers; group without regular physical activity both before and after IS, (2) exercise dropouts; regularly engaged in physical activity prior to IS, but no physical activity since diagnosis, (3) new exercisers; no regular physical activity before IS but started regular physical activity after diagnosis, and (4) exercise maintainers; maintained regular physical activity before and after IS diagnosis, based on the categorization of regular physical activity status at health checkups before and after IS diagnosis. Figure 1 presents the overall configuration and design of the study.

### Covariates and outcome definition

The main outcome of our study was the incidence of MI, which we defined as at least one hospitalization claim with the ICD-10 codes I21 or I22. Previous studies have evaluated the accuracy of this diagnostic approach, confirming its validity with a positive predictive value of 92% [27]. The date of the second health checkup was defined as the index date, and the participants were followed up until December 31, 2019, or until the development of the primary outcome, whichever occurred first.

For covariates, we gathered demographic information, including age, sex, height, weight, and waist circumference, as well as data on health-related lifestyles. These included smoking habits, categorized as either current smokers or non-smokers, and alcohol consumption, categorized as alcohol users (those who consumed any amount of alcohol) or non-users. Data on baseline comorbidities included hypertension, dyslipidemia, diabetes mellitus, and chronic kidney disease (CKD). The method for defining covariates and outcomes in cardiovascular research based on the K-NHID has been well-established in multiple studies [27–29]. The definition of obesity is a body mass index (BMI) > 25 (kg/m^2^), according to the World Health Organization recommendations for Asian populations [30]. CKD was defined as an estimated glomerular filtration rate of < 60 mL/min/1.73 m [2], calculated using the CKD epidemiology collaboration equation [32]. Participants were defined as low-income recipients when they received medical benefits and fell within the lowest income quartile. Additionally, laboratory data, such as random glucose levels, total cholesterol, glomerular filtration rate, and systolic/diastolic blood pressure, were obtained. Covariates including age, sex, smoking habits, alcohol consumption, income level, and medical history including diabetes mellitus, hypertension, dyslipidemia, and CKD were considered confounders and were adjusted for in the analysis.

### Statistical analysis

Descriptive statistics were used to examine the baseline and demographic characteristics of the participants. These were expressed as the mean ± standard deviation for continuous variables and as counts and frequencies for categorical variables. Differences in demographic and clinical attributes between the study groups were analyzed using the one-way analysis of variance for continuous variables and the chi-square test for categorical variables. MI incidence rate was calculated by dividing the number of events by 1,000 person-years (PY). Multivariable Cox proportional hazards regression models were used to estimate the adjusted hazard ratios (aHR) with 95% confidence intervals (CIs). Persistent non-exercisers were used as the reference group in the analysis. The Cox models were sequentially adjusted as follows: Model 1 for age and sex; and Model 2 for covariates of Model 1 plus other confounders, including the history of hypertension, diabetes, dyslipidemia, CKD, alcohol consumption, smoking status, and income levels. Additionally, we conducted a subgroup analysis according to age (40–65 vs. >65 years) and sex. We stratified the new exercisers into two subgroups based on MET-min/week, with a cutoff of 1,000, to identify whether the amount of physical activity was associated with the risk of MI incidence. The same regression method was used for the subgroup analysis. Additionally, we used the Kaplan-Meier method to estimate the outcome event rates across the study groups. Subsequently, the log-rank test was employed to assess the statistical significance of these rates between the groups. All statistical analyses were conducted using SAS 9.4 software (SAS Institute, Cary, NC, USA), and *p*-values < 0.05 were considered statistically significant.

## Results

### Baseline characteristics

Overall, 224,764 patients were included in this study with a mean follow-up duration of 4.25 years (mean age 65.4 years, male 50.37%). The amounts of participants in the persistent non-exerciser, exercise dropout, new exerciser, and exercise maintainer groups were 87,450, 41,245, 40,354, and 55,715 patients, respectively (Table 1). The prevalence of hypertension, diabetes, hyperlipidemia, CKD, and smoking were 64.72%, 25.18%, 54.66%, 12.7%, and 11.8%, respectively. The proportion of patients with obesity was 38.54%. The overall incident MI rate for all patients was 2.94%, and the incidence rate of MI was highest in the persistent non-exerciser group (3.51%), whereas it was lowest in the exercise maintainers group (2.09%).

**Table 1:** Demographic and clinical characteristics of study population according to the status of physical activity changes

	Total	Physical Activity				
		Persistent non-exerciser	Exercise dropouts	New exerciser	Exercise maintainers	*p*-value
*n*	224,764	87,450	41,245	40,354	55,715	
Stroke-age	64.49 ± 10.44	66.93 ± 10.19	64.99 ± 10.14	63.58 ± 10.25	60.94 ± 10.09	< 0.0001
Sex, Male	113,208(50.37)	37,019(42.33)	21,048(51.03)	20,338(50.4)	34,803(62.47)	< 0.0001
Age	65.44 ± 10.55	67.93 ± 10.29	65.95 ± 10.24	64.53 ± 10.37	61.81 ± 10.20	< 0.0001
≥ 65 years	122,513(54.51)	55,996(64.03)	23,443(56.84)	20,578(50.99)	22,496(40.38)	< 0.0001
Smoking	26,528(11.80)	10,176(11.64)	4,659(11.30)	4,899(12.14)	6,794(12.19)	< 0.0001
Drinking	55,048(24.49)	15,420(17.63)	9,041(21.92)	10,544(26.13)	20,043(35.97)	< 0.0001
Low income	43,682(19.43)	17,764(20.31)	8,047(19.51)	8,109(20.09)	9,762(17.52)	< 0.0001
Obesity (BMI ≥ 25)	86,629(38.54)	33,430(38.23)	15,880(38.50)	15,711(38.93)	21,608(38.78)	0.0538
Diabetes Mellitus	56,605(25.18)	23,140(26.46)	10,722(26.00)	9,992(24.76)	12,751(22.89)	< 0.0001
Hypertension	145,463(64.72)	59,323(67.84)	26,998(65.46)	25,727(63.75)	33,415(59.97)	< 0.0001
Dyslipidemia	122,855(54.66)	47,248(54.03)	22,755(55.17)	22,364(55.42)	30,488(54.72)	< 0.0001
Chronic kidney disease	28,541(12.70)	13,164(15.05)	5,424(13.15)	4,718(11.69)	5,235(9.40)	< 0.0001
Height(cm)	159.50 ± 9.11	157.36 ± 9.07	159.53 ± 8.87	159.79 ± 8.88	162.64 ± 8.55	< 0.0001
Weight(kg)	61.83 ± 10.67	59.95 ± 10.58	61.80 ± 10.44	62.16 ± 10.45	64.55 ± 10.52	< 0.0001
BMI	24.22 ± 3.14	24.14 ± 3.30	24.21 ± 3.13	24.27 ± 3.08	24.32 ± 2.91	< 0.0001
Waist Circumference(cm)	83.30 ± 8.65	83.30 ± 8.90	83.39 ± 8.58	83.19 ± 8.61	83.31 ± 8.32	0.0098
Serum Fasting Glucose(mg/dL)	105.44 ± 28.82	105.98 ± 30.40	105.93 ± 29.13	104.95 ± 28.03	104.59 ± 26.50	< 0.0001
Systolic BP(mmHg)	127.17 ± 15.32	127.94 ± 15.80	127.32 ± 15.43	126.85 ± 15.12	126.07 ± 14.53	< 0.0001
Diastolic BP(mmHg)	77.19 ± 9.87	77.19 ± 10.02	77.17 ± 9.90	77.21 ± 9.80	77.19 ± 9.65	0.9543
Total Cholesterol(mg/dL)	182.09 ± 41.54	183.17 ± 41.73	181.77 ± 41.66	181.75 ± 41.78	180.87 ± 40.94	< 0.0001
GFR	84.08 ± 42.74	82.71 ± 37.25	83.53 ± 41.32	84.92 ± 45.82	86.04 ± 48.98	< 0.0001
Pre-physical activity (METs)	462.29 ± 679.25	N/A	1030.83 ± 644.37	N/A	1101.83 ± 644.51	.
Post-physical activity (METs)	472.02 ± 693.40	N/A	N/A	1047.11 ± 649.95	1145.79 ± 652.30	.
**MI Event**	6,611(2.94)	3,068(3.51)	1,291(3.13)	1,086(2.69)	1,166(2.09)	< 0.0001
**F/U Duration**						
Mean ± SD	4.25 ± 2.00	4.19 ± 2.01	4.26 ± 2.01	4.33 ± 1.99	4.29 ± 1.98	< 0.0001
Median (Q1-Q3)	4.15 (2.60–5.91)	4.06 (2.55–5.84)	4.16 (2.60–5.94)	4.25 (2.68-6.00)	4.19 (2.61–5.97)	< 0.0001

Abbreviations. METs; metabolic equivalents of tasks, BP; blood pressure, MI; myocardial infarction, BMI; body mass index, GFR; glomerular filtration rate

### Incidence rates of MI in different physical activity groups

The incidence rate of MI varied among the different physical activity groups; persistent non-exercisers, exercise dropouts, new exercisers, and exercise maintainers had an incidence rate of 8.37, 7.34, 6.21, and 4.88, respectively (Table 2). Compared with persistent non-exercisers, new exercisers, and exercise maintainers had a significantly reduced risk of incident MI after adjusting for confounders including age, sex, smoking habits, alcohol consumption, income level, and history of diabetes mellitus, hypertension, dyslipidemia, and CKD (new exercisers: aHR 0.849, 95% CI: 0.792–0.911, *P*-value < 0.0001; and exercise maintainers: aHR 0.746, 95% CI: 0.696–0.801, *P*-value < 0.0001, respectively) (Table 2). The Kaplan–Meier curve for the overall population showed that the MI incidence was significantly different between persistent non-exercisers and exercise dropouts, new exercisers, and exercise maintainers (Fig. 2). The log-rank test showed a statistically significant difference (*P*-value < 0.0001).

**Table 2:** Risk for myocardial infarction after ischemic stroke according to change of physical activity

PA Change	*N*	MI	Duration	IR	Model 1		Model 2	
					HR (95% CI)	*P*-value	HR (95% CI)	*P*-value
Persistent non-exerciser	87,450	3,068	366,562.45	8.37	1(Ref.)		1(Ref.)	
Exercise dropouts	41,245	1,291	175,807.81	7.34	0.923(0.865,0.986)	0.0166	0.937(0.878,1.000)	0.0507
New exercisers	40,354	1,086	174,738.83	6.21	0.831(0.775,0.891)	< 0.0001	0.849(0.792,0.911)	< 0.0001
Exercise maintainers	55,715	1,166	239,042.63	4.88	0.710(0.662,0.761)	< 0.0001	0.746(0.696,0.801)	< 0.0001

Abbreviations. MI; myocardial infarction, IR; incidence rate, HR; hazard ratio, CI; confidence interval, PA; physical activity

#Incidence rate: dividing the number of events by 1,000 person-years

#Model 1: age and sex-adjusted, Model 2: Model 1 + alcohol intake, smoking status, economic status, history of hypertension, diabetes, dyslipidemia, and chronic kidney disease

**Fig. 2:**
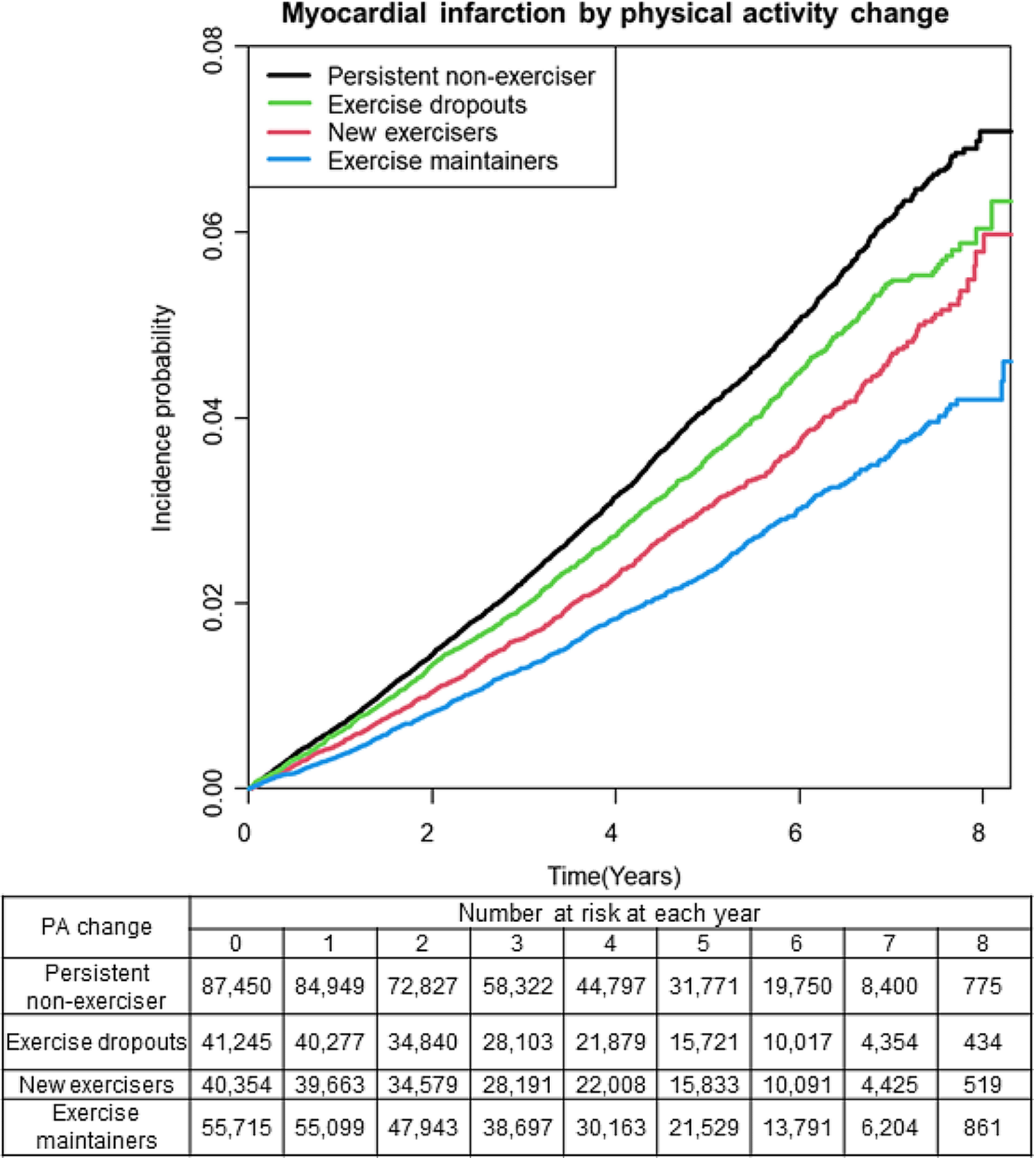
Incidence probability of myocardial infarction after ischemic stroke according to change of physical activity habits. (Note this analysis was adjusted for age, sex, smoking, alcohol intake, economic status, history of hypertension, diabetes, dyslipidemia, and chronic kidney disease.)

### Subgroup analysis

We performed a subgroup analysis to determine whether the association between changes in physical activity and the risk of MI was affected by age and sex. As in the primary analysis, consistent findings were observed in both the male and female subgroups (except for female exercise dropouts). When divided into those aged over 65 years or not, only those who continued physical activity showed statistical significance in the < 65 years group. (aHR 0.811, 95% CI: 0.723–0.910, *P*-value = 0.0004). However, within the specific age group of 65 years and older, similar to the primary analysis, a decrease in MI was observed in all groups that had engaged in any form of physical activity in comparison to those who were persistent non-exercisers (exercise dropouts aHR 0.925, 95% CI 0.857–0.998, *P*-value = 0.0442; new exercisers aHR 0.839, 95% CI: 0.772–0.912, *P*-value < 0.0001; exercise maintainers aHR 0.713, 95% CI: 0.652–0.779, *P*-value < 0.0001) (Table 3).

**Table 3:** Subgroup analysis according to the age and sex group and risk for myocardial infarction after ischemic stroke

						Model 1		Model 2	
	Physical activity	*N*	MI	Duration	IR	HR (95% CI)	*P*-value	HR (95% CI)	*P*-value
Male	Persistent non-exerciser	37,019	1447	149206.62	9.70	1(Ref.)		1(Ref.)	
	Exercise dropouts	21,048	694	86966.98	7.98	0.868(0.793,0.951)	0.0022	0.879(0.802,0.962)	0.0051
	New exerciser	20,338	592	86179.98	6.87	0.801(0.728,0.881)	< 0.0001	0.815(0.740,0.897)	< 0.0001
	Exercise maintainers	34,803	810	148384.43	5.46	0.711(0.652,0.776)	< 0.0001	0.746(0.683,0.814)	< 0.0001
Female	Persistent non-exerciser	50,431	1621	217355.82	7.46	1(Ref.)		1(Ref.)	
	Exercise dropouts	20,197	597	88840.83	6.72	0.989(0.900,1.087)	0.8197	1.007(0.916,1.106)	0.8925
	New exerciser	20,016	494	88558.85	5.58	0.863(0.780,0.955)	0.0044	0.887(0.802,0.982)	0.0206
	Exercise maintainers	20,912	356	90658.20	3.93	0.691(0.615,0.776)	< 0.0001	0.728(0.649,0.818)	< 0.0001
*p* for interaction						0.1505		0.1245	
40–64 yrs	Persistent non-exerciser	31,454	673	139425.79	4.83	1(Ref.)		1(Ref.)	
	Exercise dropouts	17,802	367	79071.84	4.64	0.958(0.844,1.088)	0.5093	0.980(0.863,1.113)	0.7576
	New exerciser	19,776	362	88759.91	4.08	0.858(0.755,0.975)	0.0190	0.885(0.778,1.005)	0.0606
	Exercise maintainers	33,219	524	145805.68	3.59	0.764(0.681,0.856)	< 0.0001	0.811(0.723,0.910)	0.0004
≥ 65 yrs	Persistent non-exerciser	55,996	2395	227136.65	10.54	1(Ref.)		1(Ref.)	
	Exercise dropouts	23,443	924	96735.97	9.55	0.913(0.846,0.986)	0.0199	0.925(0.857,0.998)	0.0442
	New exerciser	20,578	724	85978.93	8.42	0.823(0.757,0.895)	< 0.0001	0.839(0.772,0.912)	< 0.0001
	Exercise maintainers	22,496	642	93236.95	6.89	0.682(0.624,0.745)	< 0.0001	0.713(0.652,0.779)	< 0.0001
*p* for interaction						0.4903		0.3701	

Abbreviations. MI; myocardial infarction, IR; incidence rate, HR; hazard ratio, CI; confidence interval

#Incidence rate: dividing the number of events by 1,000 person-years

#Model 1: age and sex-adjusted, Model 2: Model 1 + alcohol intake, smoking status, economic status, history of hypertension, diabetes, dyslipidemia, and chronic kidney disease

Irrespective of factors like smoking, alcohol use, income status, hypertension, diabetes, dyslipidemia, or CKD, modifications in physical activity yielded outcomes consistent with the primary analysis regarding the occurrence of MI in patients following an IS. Further subgroup analyses were conducted to assess whether the relationship between changes in physical activity patterns and the risk of MI following IS was influenced by the intensity of physical activity. Regardless of pre-stroke physical activity, exercising > 1,000 MET-min/week after IS was consistently associated with a lower risk of MI (Supplementary Tables 1,2).

## Discussion

In this nationwide population-based cohort study, we identified several key findings. First, both the commencement and continuation of regular physical activity following IS diagnosis were significantly associated with a decreased risk of MI, a trend that remained consistent even among exercise dropouts. Importantly, this trend was observed consistently across both sexes and in age groups both above and below 65 years, independent of the presence of vascular risk factors.

### Physical activity change and MI after IS

This study demonstrated that initiating routine physical activity may reduce the risk of MI following an IS in individuals who previously led a sedentary lifestyle. Similarly, active individuals should maintain their physical activity. Even if someone experiences a stroke and faces limitations in physical activity, those who previously engaged in physical activity still demonstrated improved outcomes, emphasizing the importance of regular physical activity.

In subgroup analysis, both males and females (excluding female exercise dropouts) who engaged in any form of physical activity were associated with a lower incidence of MI than those who did not physical activity. This trend aligns with the main analysis in which exercise dropouts had the highest MI incidence rates, followed by new exercisers and exercise maintainers. All types of physical activity, regardless of both mild (MET-min/week < 1,000) and moderate-to-intense (MET-min/week ≥ 1,000) physical activity, demonstrated the same significance in reducing the incidence of MI after IS. This finding further highlights the consistent and crucial role of physical activity in reducing the risk of MI after IS, irrespective of the intensity or level of physical activity and age. A study that examined the relationship between sedentary behavior, physical activity, and the incidence of IS and MI showed that individuals with less sedentary time and more physical activity had a lower incidence of both IS and MI, even among those over 70 years of age [14]. These findings serve as an urgent reminder, emphasizing the importance of physical activity for preventing MI after IS, particularly among individuals aged 65 years and older, as the population ages and the growth of “old man-living young” groups engage in more physical activity. In a Korean survey study evaluating the exercise habits of community-dwelling stroke patients, it was found that 67% perform some level of physical activity. Among these, while more than half exercised daily, over 88% engaged only in low-intensity exercises [31]. Given our findings that moderate to vigorous intensity exercise is associated with a reduced risk of myocardial infarction (MI) after stroke, there is a clear justification for promoting more intense physical activities in this population.

### Mechanism for the benefits of physical activity in MI after IS

Regular physical activity may reduce the risk of developing various cardiovascular diseases [32] after IS by lowering blood pressure and cholesterol [33], improving glucose tolerance [34], and enhancing arterial function [35]. This mechanism can be attributed to how regular physical activity effectively reduces blood pressure [36]. Increasing physical activity after IS reduces MI and all-cause mortality as well as contributes to the secondary prevention of subsequent stroke [37, 38]. Numerous studies have investigated the preventive effects of physical activity against IS and MI. Some studies have reported a lack of association between physical activity and the risk of MI or IS or demonstrated an association only in specific sexes [39, 40]. Previous studies examining the relationship between physical activity and the risk of MI or IS have focused on each disease individually, often presenting inconsistent findings. Conversely, our study possesses particular significance as it delivers an optimistic finding that any form of physical activity, irrespective of sex or intensity, is beneficial compared to a sedentary lifestyle for older adults. By emphasizing the importance of regular physical activity for prevention, our study contributes to the broader goal of encouraging individuals to engage in physical activity. This finding carries both medical significance and social relevance, as it promotes the understanding that any level of physical activity is better than none in preventing adverse health outcomes in older adults.

It is important to recognize that patients who were not regularly active before experiencing a stroke may find it challenging to initiate physical activity afterward, potentially introducing bias into the study. This study included patients with mild ambulatory stroke who participated in a survey to address these challenges. Additionally, even when considering the level of physical activity intensity measured using the METs, the group that engaged in more vigorous physical activity exhibited better outcomes. These findings indicate that the role of physical activity after stroke remains significant. This study underscores the significance of physical activity in stroke survivors receiving outpatient care, particularly in those who can actively engage in physical activity.

### Strength and limitations

This study had some limitations. First, because this study relied on claims data from a population-based cohort study, important clinical variables were unavailable in the stroke population data. These variables included factors such as stroke severity, medications prescribed at discharge (including antithrombotics and statins), and an individual’s history of conditions such as atrial fibrillation, congestive heart failure, or peripheral vascular disease, all of which are recognized as risk factors for MI after IS [2, 41]. The absence of data on stroke severity and specific post-stroke treatments may bias our findings towards a protective effect of physical activity. Patients with milder strokes, who are more likely to engage in physical activity, inherently have a lower risk of subsequent MI, which could overestimate the protective effect attributed to physical activity. Moreover, not accounting for the protective impact of prescribed medications could further skew the results, attributing benefits to physical activity that might be partially due to differences medication adherence. Second, relying solely on ICD-10 codes and hospitalization records to define MI may be prone to coding errors. Additionally, this coding scheme does not distinguish between type 1 and type 2 non-ST-elevation MI, which have differing risk factors and prognoses. Consequently, our findings may not accurately differentiate the impact of physical activity on each type of MI. Third, there is a possibility of bias caused by changes in the participants’ physical activity between the initial assessment and the end of the follow-up period. However, as the dataset in our study grew, the average values for each group eventually converged. Despite the potential variation in the number of individuals within each group over time, we anticipate that the statistical significance of our findings will remain consistent. Fourth, the assessment of physical activity relied solely on patient self-reporting. The questions used for this assessment may have had limitations regarding quality and may not have accurately reflected the actual level of physical activity. Additionally, physical activity was not subdivided into specific categories such as work-related physical activity, leisure-time physical activity, or participation in sports. Instead, it was treated as a single entity, which may limit our ability to analyze the potential differential effects of various types of physical activity on the incidence of MI after IS. Furthermore, self-reporting bias may have led to an overestimation of the effects of physical activity on MI incidence. This is particularly evident as individuals who are generally healthier and more conscious of their vascular health might tend to overreport their levels of physical activity. Such overreporting could falsely amplify the perceived protective effects of physical activity against MI, suggesting a stronger benefit than might actually exist. This bias is crucial to consider, as it may skew our understanding of the true impact of lifestyle factors on heart health. Finally, since our study participants only included those able to attend ambulatory national health checkups and complete self-reported questionnaires, it is likely that they experienced milder forms of ischemic stroke. Therefore, our results might not be applicable to the entire stroke population. Despite these limitations, this large-scale nationwide study offers valuable and novel evidence by capturing the level of physical activity before and after stroke diagnosis, unlike previous literature on the participant.

## Conclusion

In this retrospective study that encompassed the entire Korean population, we observed that starting or continuing a regular physical activity after being diagnosed with IS was associated with a decreased incidence of MI compared to individuals who did not engage in any physical activity. The association remained consistent across different age groups and is unaffected by sex or the presence of vascular risk factors. This emphasizes the significance of promoting physical activity as an essential aspect of post-stroke care in older adults, as it may reduce the risk of MI. Given the observational nature of our study, further clinical trials are warranted to rigorously assess the efficacy and practical implications of physical activity promotion in IS survivors.

### Electronic supplementary material

Below is the link to the electronic supplementary material.

Supplementary Material 1

## Data Availability

The anonymized dataset for this study is publicly available from the Korean National Health Insurance Sharing Service and can be accessed at https://nhiss.nhis.or.kr/bd/ab/bdaba000eng.do.
