# Supplementary material for: Association between physical activity changes and incident myocardial infarction after ischemic stroke: a nationwide population-based study
Source: BMC Public Health. 2024 May 6;24:1241. doi: 10.1186/s12889-024-18724-2 (PMC11071236; doi:10.1186/s12889-024-18724-2)
Supplement: Supplementary file 1 — Supplementary Material 1 [file 12889_2024_18724_MOESM1_ESM.docx]

**Supplementary Table 1. Subgroup analysis according to the comorbidities and life style behaviors and risk for myocardial infarction after ischemic stroke**

| Subgroup | | HR (95% CI) | | | | *p* for interaction |
| --- | --- | --- | --- | --- | --- | --- |
|  |  | Persistent non-exerciser | Exercise dropouts | New exercisers | Exercise maintainers |  |
| Smoking | No | 1(Ref.) | 0.943(0.880,1.012) | 0.831(0.770,0.897) | 0.748(0.694,0.806) | 0.3726 |
|  | Yes | 1(Ref.) | 0.894(0.746,1.070) | 0.954(0.801,1.136) | 0.739(0.617,0.884) |  |
| Alcohol intake | No | 1(Ref.) | 0.972(0.905,1.045) | 0.878(0.812,0.950) | 0.764(0.704,0.829) | 0.0429 |
|  | Yes | 1(Ref.) | 0.782(0.667,0.915) | 0.732(0.627,0.855) | 0.675(0.589,0.773) |  |
| Income | Other | 1(Ref.) | 0.921(0.856,0.991) | 0.832(0.769,0.900) | 0.742(0.687,0.802) | 0.6051 |
|  | Low Q1 | 1(Ref.) | 1.003(0.869,1.157) | 0.917(0.789,1.065) | 0.764(0.655,0.892) |  |
| DM | No | 1(Ref.) | 0.921(0.850,0.999) | 0.859(0.788,0.935) | 0.734(0.674,0.799) | 0.7089 |
|  | Yes | 1(Ref.) | 0.966(0.866,1.078) | 0.830(0.736,0.936) | 0.772(0.687,0.868) |  |
| Hypertension | No | 1(Ref.) | 0.991(0.872,1.126) | 0.874(0.763,1.000) | 0.750(0.659,0.854) | 0.7758 |
|  | Yes | 1(Ref.) | 0.919(0.852,0.992) | 0.841(0.775,0.912) | 0.746(0.688,0.810) |  |
| Dyslipidemia | No | 1(Ref.) | 0.913(0.828,1.005) | 0.816(0.735,0.905) | 0.749(0.677,0.829) | 0.6790 |
|  | Yes | 1(Ref.) | 0.958(0.877,1.046) | 0.878(0.799,0.964) | 0.745(0.678,0.818) |  |
| CKD | No | 1(Ref.) | 0.953(0.886,1.026) | 0.844(0.780,0.913) | 0.739(0.684,0.799) | 0.5496 |
|  | Yes | 1(Ref.) | 0.877(0.762,1.011) | 0.870(0.748,1.012) | 0.782(0.669,0.914) |  |
| \| Abbreviations. DM; diabetes mellitus, CKD; chronic kidney disease, HR; hazard ratio, CI; confidence interval, Low Q1; income level quartile 1 \| \| --- \| \| #Model only adjusted for age, sex, smoking status, alcohol intake, economic status, history of hypertension, diabetes, dyslipidemia, and chronic kidney disease \| | | | | | | |

**Supplementary Table 2. Subgroup analysis according to the intensity of physical activity and risk for myocardial infarction after ischemic stroke**

| Pre-METS | Post-METS | No. | MI | Duration | IR | Model 1  HR (95% CI) | Model 2  HR (95% CI) |
| --- | --- | --- | --- | --- | --- | --- | --- |
| No physical activity | No physical activity | 87450 | 3068 | 366562.45 | 8.37 | 1(Ref.) | 1(Ref.) |
|  | METS <1000 | 22838 | 623 | 98709.03 | 6.31 | 0.863(0.792,0.941) | 0.887(0.814,0.967) |
|  | METS ≥1000 | 17516 | 463 | 76029.80 | 6.09 | 0.790(0.716,0.872) | 0.803(0.727,0.885) |
| METS <1000/week | No physical activity | 23641 | 709 | 101328.75 | 7.00 | 0.912(0.840,0.990) | 0.928(0.855,1.007) |
|  | METS <1000 | 17467 | 349 | 75138.99 | 4.64 | 0.740(0.661,0.828) | 0.784(0.700,0.878) |
|  | METS ≥1000 | 11569 | 207 | 50068.88 | 4.13 | 0.613(0.532,0.706) | 0.642(0.557,0.740) |
| METS ≥1000/week | No physical activity | 17604 | 582 | 74479.06 | 7.81 | 0.937(0.857,1.024) | 0.947(0.867,1.036) |
|  | METS <1000 | 9922 | 223 | 42980.86 | 5.19 | 0.724(0.632,0.830) | 0.752(0.656,0.863) |
|  | METS ≥1000 | 16757 | 387 | 70853.90 | 5.46 | 0.737(0.662,0.820) | 0.777(0.698,0.865) |
| Abbreviations. METs; metabolic equivalents of tasks, MI; myocardial infarction, IR; incidence rate, HR; hazard ratio  #Incidence rate: dividing the number of events by 1,000 person-years  #Model 1: age and sex-adjusted, Model 2: Model 1 + smoking status, alcohol intake, economic status, history of hypertension, diabetes, dyslipidemia, and chronic kidney disease | | | | | | | |
